# Microbial Contamination of Glaucoma Eyedrops Used by Patients Compared With Ocular Medications Used in the Hospital

**DOI:** 10.1097/MD.0000000000000583

**Published:** 2015-02-27

**Authors:** Barbara Teuchner, Julia Wagner, Nikolaos E. Bechrakis, Dorothea Orth-Höller, Markus Nagl

**Affiliations:** From the Department of Ophthalmology (BT, JW, NEB); and Department of Hygiene, Microbiology and Social Medicine (JW, DO-H, MN), Division of Hygiene and Medical Microbiology, Medical University of Innsbruck, Innsbruck, Austria.

## Abstract

The aim of this study was to compare the percentage of contamination of multiuse eyedrops applied by glaucoma patients at home and by the medical personnel at the outpatient department, the ward, and the operating room of our Department of Ophthalmology.

Eyedrops were collected over a period of 11 months. Samples were taken from the dropper tip (smear), drops, and the residual fluid inside the bottle and cultivated on blood agar. Colony forming units were counted and identified by mass spectrometry.

The percentage of contamination was significantly higher in eyedrops applied by the patients (29/119; 24.4%, *P* < 0.01), used in the ward (26/133; 19.5%, *P* < 0.01), and in the outpatient unit (6/35; 17.1%, *P* = 0.036) compared with that in the operating room (6/113; 5.3%). The median period of use was 1 week in the operating room compared with 4 weeks in the other groups (*P* < 0.01). Glaucoma medications were significantly more frequently contaminated than antibiotic and anesthetic eyedrops (*P* < 0.05). For eyedrops applied by the patients, the tip was more frequently contaminated than the drops and the residual internal fluid. For eyedrops from the ward, the opposite was true. Pathogenic strains (*Pseudomonas aeruginosa, Serratia marcescens, Acinetobacter lwoffii*, *Stenotrophomonas maltophilia,* and *Staphylococcus aureus*) were found only in 6 bottles (1.5%), whereas most of the detected microbes belonged to human or environmental flora.

This study underlines the importance of hygienic handling of eyedrops and raises the question of whether single-use glaucoma medication might be preferred to reduce the risk of contamination.

## INTRODUCTION

Ophthalmic solutions may become contaminated with microorganisms during repeated use.^1–5^ The percentage of contamination in studies shows an enormous variation between 0.07% and up to 70%.^3,6,7^ It is generally accepted that the contamination increases with the period of application of the drops,^1,6,8^ although not all studies are in agreement on this point.^9,10^ Also, the population of patients is proposed to have an influence, particularly in the case of improper use.^4^ There were hints for a lower percentage in the operating room compared with the outpatient department and the ward in the investigation of Nentwich et al,^3^ but the difference was not significant, probably because of the small sample size. No difference between the frequency of contamination (overall 16.3%) in an ophthalmic department and in a nursing home was found.^11^

Pathogens grown from eyedrops flasks have been mainly representatives of the skin flora and the environment,^3,4,6,9,11–13^ but also, with a low frequency, *Staphylococcus aureus* and gram-negative rods like *Pseudomonas aeruginosa*,^1,4,8,14,15^*Proteus mirabilis*,^2,15^ and *Serratia marcescens*.^15^ Although the contamination of eyedrops seems not to cause frequent inflammation and preservatives should inactivate pathogens in the residual fluid, keratitis,^15–18^ conjunctivitis,^4,19^ and even endophthalmitis^5^ have been reported to be caused by the latter bacteria cultivated from the applied eyedrops. There is a risk for these infections, particularly in the case of disrupted epithelium such as abrasion or leaking filtering blebs.

Of note, the location of sampling from eyedrops bottles (drops, residual fluid, and cap/tip) plays a role in that; generally, the tip was the most frequently contaminated part.^1,3,5,9^ On the contrary, in single studies, the drops and the residual fluid were most frequently contaminated.^6,11^

To contribute to clarification of some of the abovementioned uncertainties, we performed this study at our department, the Department of Ophthalmology in Innsbruck, Austria. The first aim was to compare the percentage of contamination of multiuse eyedrops used by glaucoma patients without signs of conjunctivitis and multiuse ocular medications used by the medical staff at the outpatient department, the ward, and the operating room. Second, we wanted to test the influence of sampling from the eyedrops tips, the drops, and the residual fluid inside the bottle.

## MATERIALS AND METHODS

### Ophthalmic Medications

Eyedrops were collected during a period of 11 months from the operating room, the ward, and the outpatient department of the Department of Ophthalmology of the Medical University of Innsbruck. These topical ocular medications were applied to patients only by nurses and physicians. Chronic glaucoma patients from our glaucoma outpatient department were asked to bring their eyedrops bottles after ∼4 weeks of use to the clinical examination. Only multiuse topical ocular medications were included, and the date of first use was noted on their label. There were no restrictions regarding either the drug or disease. The period of use was recorded. Eyedrops were stored for a maximum of 24 hours at 4°C before sampling.

The study was performed in accordance with the Declaration of Helsinki, and informed consent was obtained from all patients who gave their drops for the study. Ethical approval was not necessary because all eyedrops bottles were collected anonymously.

### Sample Taking

Samples were taken from the tip of the bottles, the drops, and the residual fluid inside the bottles. First, the cap was removed, and the tip was smeared onto Columbia agar containing 5% sheep blood (Heipha Diagnostika, Eppelheim, Germany), starting with 1 line over the middle of the plate followed by smearing over the whole agar area at a right angle 2 times.

Subsequently, 4 drops were put into sterile 1.7 mL tubes (VWR, Vienna, Austria). Aliquots of 100 μL were pipetted on Columbia blood agar and spread with a platin loop. Lastly, residual fluid was aspirated with a 27 gauge needle into a 1 mL syringe via the dropping canal. If this was not possible because of very little residual fluid or improper size of the flask, the surface of the flask was disinfected with Mikrozid (Schülke & Mayr, Vienna, Austria) and penetrated with the needle to obtain the fluid. As described above, 100 μL of the fluid was spread on agar plates.

All plates were incubated at 37°C under aerobic conditions for 48 hours. Subsequently, they were observed for growth of bacteria or fungi, and colony forming units (CFUs) were counted. Representative colonies were subcultivated on Columbia blood agar to gain pure cultures after 48 hours of growth. Aliquots from these were deep frozen in 10% skim milk for storage.^20^

### Identification of Microorganisms

Bacteria and fungi stored in skim milk were cultivated on Columbia blood agar. A Gram stain was performed. Subsequent analysis was done by matrix-assisted laser desorption/ionization time-of-flight (MALDI-TOF) mass spectrometry using a Microflex MALDI-TOF mass spectrometer (Bruker Daltonics GmbH, Bremen, Germany). The direct smear method on the target plate was applied, and α-cyano-4-hydroxycinnamic acid (1 μL) was added as a matrix (according to the instructions by Bruker Daltonics). The spectrum was analyzed with the respective MALDI Biotyper 3.0 software and library system 3.1.0 (Bruker Daltonics). According to the manufacturer's recommendations, a score of ≥2.000 was set for identification of the species, and a score between 1.700 and 1.999 for identification of the genus. Fungi were also determined morphologically after staining with lactophenol blue. Additionally, oxidase testing was performed to enhance the diagnostic reliability of *P aeruginosa* using Taxo discs (Becton, Dickinson & Company, Franklin Lakes, NJ) and optochin testing to differentiate *Streptococcus pneumoniae* from other streptococci.

### Statistics

For sample size calculation, we assumed a contamination rate of eyedrops used by the patients at home of 20%, according to previous literature.^5,8,11^ An estimation of the rate in the operating room was more difficult. Taking into account the shorter period of use and higher hygienic standards,^1,3,6^ we chose a rate of 5% in this unit. With a test power of 90%, this difference would become significant with a probability of 95% (*P* < 0.05) if 113 samples were included. Therefore, this was our limit of samples from patients at home and the operating theater, and as many samples as possible from the ward and the outpatient unit were collected in parallel.

Fisher exact test and χ^2^ test were used to test for statistical significance of frequencies of occurrence of contamination of the eyedrops between all groups (the glaucoma patients and the 3 sites). Kruskal–Wallis test was applied to compare quantitative results of the 4 groups. *P* < 0.05 was considered significant. Calculations were done with GraphPad Prism Software Inc. version 5.02 (La Jolla, CA).

## RESULTS

Overall, 400 eyedrops bottles were collected: 119 from 96 glaucoma patients, 133 from the ward, 35 from the outpatient unit, and 113 from the operating room. Medications from the glaucoma patients comprised mainly dorzolamide plus timolol, latanoprost, brimonidine, latanoprost plus timolol, travoprost, brinzolamide plus timolol, and bimatoprost; medications from the ward and outpatient unit comprised mainly mydriatics (epinephrine, tropicamide, cylcopentolate, and scopolamine), anesthetics (oxybuprocaine ± fluorescein), antiphlogistics (dexamethasone plus gentamicin, and diclofenac), and antibiotics (gentamicin, tobramycin, and ofloxacin); medications from the operating room comprised mydriatics (epinephrine, tropicamide, cylcopentolate, and atropine), anesthetics (oxybuprocaine), antibiotics (tobramycin and ofloxacin), antiphlogistics (dexamethasone plus gentamicin), and glaucoma medications (betaxolol).

The period of use, quoted as median (25% percentile, 75% percentile; minimum, maximum), was 4 (3, 4; 1, 24) weeks for the glaucoma patients, 4 (4, 4; 1, 6) weeks for the ward, 4 (4, 4; 1, 7) weeks for the outpatient unit, and 1 (1, 1; 0.29, 3.7) week for the operating room (*P* < 0.01 of the operating room vs home, ward, and outpatient unit; *P* > 0.1 between the other units, Kruskal–Wallis test).

The different topical glaucoma eyedrops from the patients used at home and the different topical preparations used in the Department of Ophthalmology are listed in Table 1. The frequency of contamination was significantly higher in the glaucoma eyedrops applied by the patients (24.4%), in the ocular medication used in the ward (19.5%), and in the eyedrops from the outpatient unit (17.1%) compared with that of the operating room (5.3%). The differences between the drops used at home, in the ward, and the outpatient unit were small and not statistically significant.

**TABLE 1:**
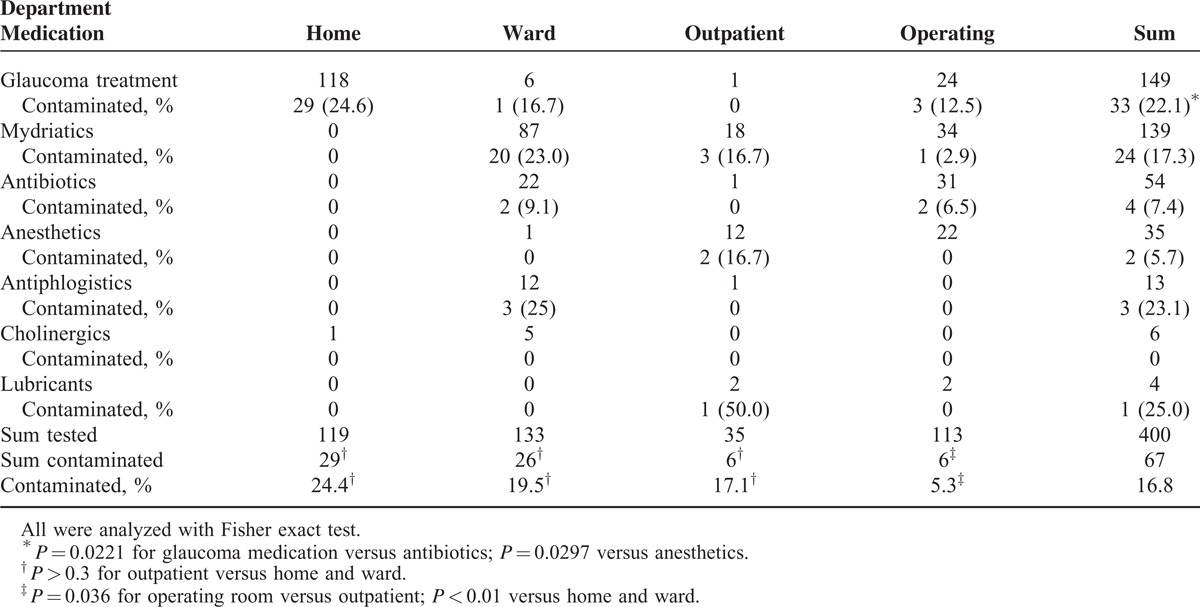
Number of Medications Tested and Number of Contaminated Ones

Glaucoma medications were significantly more frequently contaminated than antibiotic and anesthetic eyedrops (*P* < 0.05), but there were no differences between all other medications, or the number of samples was too low to allow a reasonable calculation (cholinergics and lubricants) (Table 1). Particularly striking were eyedrops containing oxybuprocaine (n = 29) and dexamethasone/gentamicin (n = 14), of which all tested bottles were sterile.

The influence of the site of sample taking is illustrated in Table 2. The residual volume was too low to evaluate the drops and residual fluid of 8 bottles in total. These dropouts did not influence the outcome, and intention-to-treat analysis was performed. Remarkably, only in 4 of the 67 contaminated bottles all sites (the bottle tip, the drops, and the residual fluid) were positive. These were exactly those containing gram-negative human pathogens, that is, *P aeruginosa* and *Pseudomonas spp*, *S marcescens*, and *Stenotrophomonas maltophilia*. In no >5 further bottles were 2 sites positive, so that in the majority of samples the contamination could be found only in 1 site of the bottle. For glaucoma eyedrops applied by the patients, the tip was more frequently contaminated (24/119, 20.2%) than the drops (10/119, 8.4%) and the residual internal fluid (6/119, 5.0%). For drops from the ward, the opposite was true. The frequencies for the tip (2/133, 1.5%) were lower than those for the drops (15/133, 11.3%) and residual fluid (10/133, 7.5%). In the outpatient and operating unit, such differences were not seen (Table 2).

**TABLE 2:**
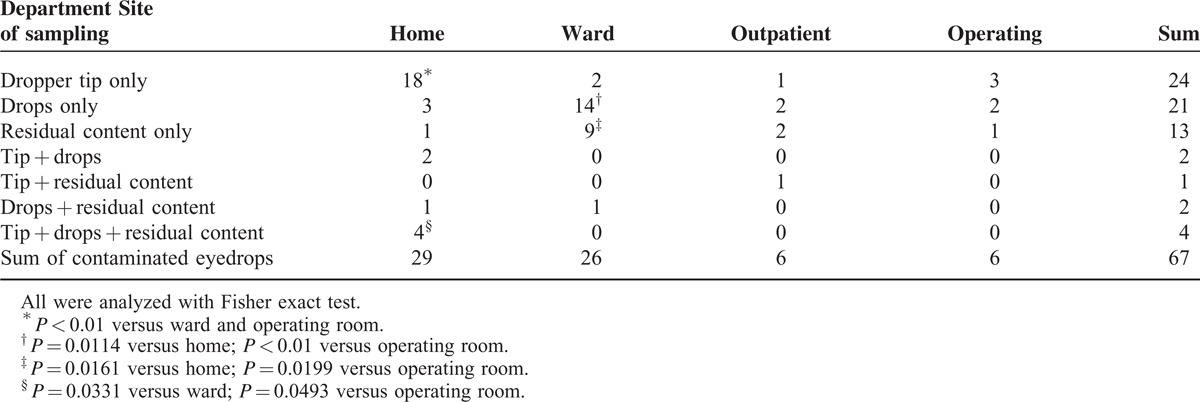
Number and Distribution of Contaminated Samples of Eyedrops

In the majority of contaminated eyedrops (53/67, 79.1%), 1 pathogen was cultivated, whereas 2 pathogens were found in 11 (16.4%) and 3 pathogens in 3 (4.5%) bottles. Species of 20 genera of bacteria could be identified (Table 3). Representatives of the human flora were prevalent, followed by environmental bacteria and also some human pathogens (Table 3). The ratio of human to environmental flora was 24:7 in samples from the bottle tip, whereas it was 5:10 in residual fluid and balanced (12:14) in the drops.

**TABLE 3:**
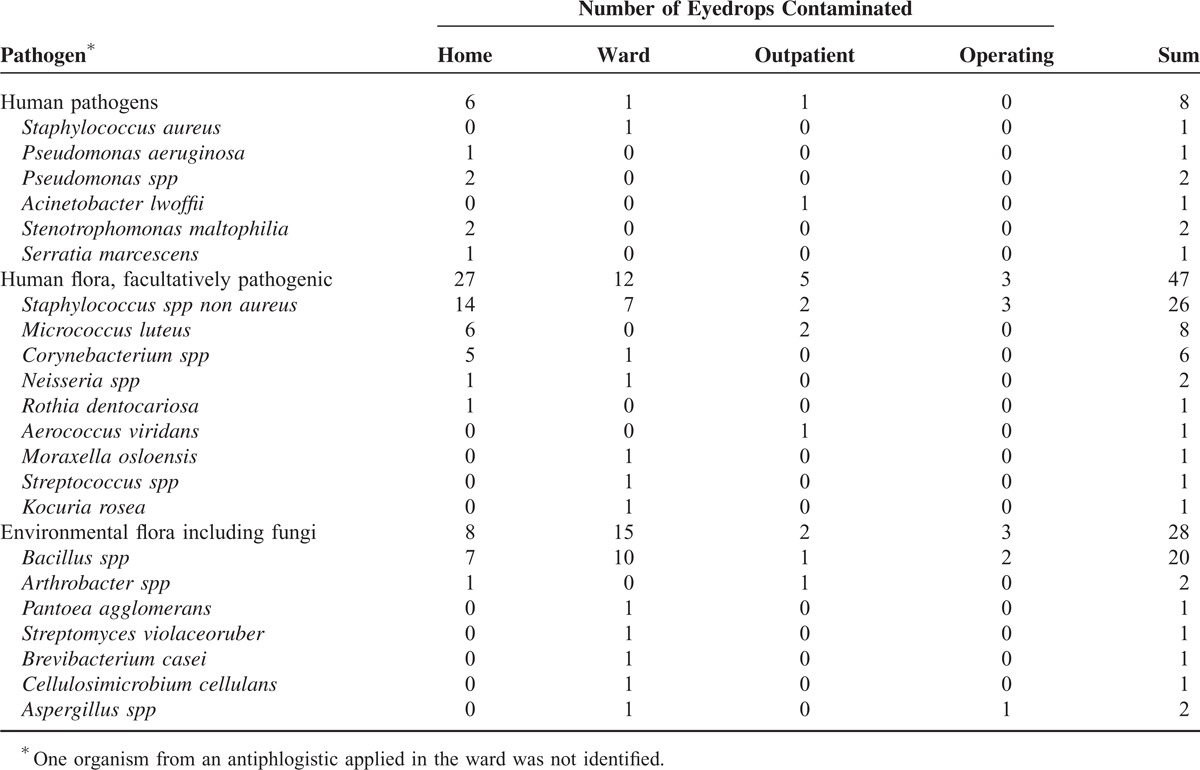
Pathogens Found in Eyedrops

Except for 1 sample from the ward (*S aureus*) and from the outpatient department (*Acinetobacter lwoffii*), the human pathogens *P aeruginosa* and *Pseudomonas spp*, *S marcescens*, and *S maltophilia* were all detected in samples from 4 bottles used by the glaucoma patients, including 1 bottle containing both *P aeruginosa* and *S marcescens*. Remarkably, these gram-negative pathogens grew from all test sites from the respective eyedrops (Tables 2 and 3). Furthermore, it was exactly in these samples that the CFU on the agar plates exceeded 1000 at least from 1 sample site and 100 from all 3 sites (tip, drops, and residual fluid). In 1 more sample from the tip of a bottle used by a glaucoma patient, *Staphylococcus xylosus* was found with >1000 CFU. In further 4 samples from eyedrops used at home, the number of CFU ranged between 56 and 300, between 2 and 6 in 7 samples, and 1 in the remaining positive samples. The highest CFU counts from the ward ranged between 2 and 6 in 5 samples, and from the outpatient unit 10 CFU grew in 1 sample. The maximum from the operating room was only 1 CFU. The frequency of samples with >50 CFU was significantly higher at home (9/119) compared with the ward and the operating unit (both *P* < 0.01).

## DISCUSSION

The average percentage of contamination of eyedrops in this study (16.8%) resembles the average of other studies,^5,8,11^ for instance. Particularly low occurrences are usually found after short application periods,^7,13^ whereas high ones may result from longer use and/or nonoptimal handling of the drops.^6,12^

One result of the present study, that the frequency of contamination was significantly lower in the operating room (5.3%), may be explained by the following facts. First, the application period of the drops was significantly shorter in this unit (1 week) than in the other groups (median of 4 weeks). Although the influence of the duration of use is accepted in general, differences in the outcome between short periods of use are controversial.^6,9,10^ Possibly, the shorter duration of use was the decisive factor in favor of the operating room, and it is difficult to draw conclusions on the following 2 points, though they are near at hand. Second, hygienic provisions are more restrictive in the operating unit, comprising both cleaning of surfaces and personnel hygiene. Third, room cleanliness is higher and, therefore, the load of environmental bacteria is reduced. Notably, the occurrence of contamination was lower in all eyedrops (particularly antibiotics, mydriatics, and glaucoma medications) independent of their content (Table 1).

We are aware that the use of different kinds of eyedrops in our investigation excluded a direct comparison of the same medication in the tested units. This limitation had to be accepted to complete the study within reasonable time at our university. Notably, the aim of the study was a comparison of different units and the sampling sites and not of special kinds of medication.

Regarding the site of contamination, the tip of the drops was prevalent in several studies,^1,3,5,9^ although a maximum of 2 sites have been compared most of the time. The results from the medications of glaucoma patients with a 24.4% frequency of contamination are in agreement with these findings (Table 2). This indicates frequent contact with the eyelids and the conjunctiva upon dosing, which is confirmed by the predominating skin and conjunctival flora found in this group. However, in eyedrops used in the ward, the results were opposite, that is, a significantly more frequent contamination of the drops and the residual fluid. An explanation may be that the eyedrops in the ward were often stored open, without the cap placed on the bottle, so that entrance of bacteria was possible. This is supported by the fact that the bacterial species found in the samples from this unit included twice as many environmental pathogens as samples from glaucoma patients. Considering that bacterial spores survive the preservatives used in eyedrops, it seems logical that *Bacillus spp* predominated in the drops and residual fluid. The number of positive samples from the outpatient and operating unit was too small to allow conclusions in this regard. As a consequence, clinical staff and patients should take care not to leave open multiuse eyedrops bottles.

As in many previous studies,^3,4,6,8,9,11,12^ human pathogens not belonging to the skin, conjunctival, or environmental flora were rare in our investigation and found only in 4 eyedrops from glaucoma patients (3 *Pseudomonas spp*, 1 *S marcescens*, and 2 *S maltophilia*) and in 1 each from the ward (*S aureus*) and outpatient department (*A lwoffii*). The species represented those well known to contaminate eyedrops. Also in accordance with previous studies,^1,5^ the gram-negatives (*P aeruginosa*, *S marcescens*, and *S maltophilia*) were grown from all sites of the bottle, whereas this was not the case for *Staphylococcus spp*, which—in the group of glaucoma patients—were predominantly isolated from the tip. This fits the assumption that gram-negative bacteria may transmigrate from the bottle tip to the interior.^5,15,16^ The low percentage of contamination with such pathogenic strains (6/400, which equals 1.5%) confirms the results of previous studies and may indicate a generally high safety of this kind of medication (for instance^1,3–5^). Nevertheless, infections from eyedrops have been reported,^4,15–19^ and particularly detection of the same pathogen from different sites of the eyedrops bottle seems to be meaningful. Notably, a high count of CFU was found only in few eyedrops applied by glaucoma patients, and the highest numbers (>1000 CFU) comprised exactly the previously mentioned gram-negative bacteria. Taking together the facts mentioned in this paragraph, eyedrops from which human pathogens can be detected at high numbers from different sites indicate a risk of infection. Therefore, we recommend taking samples from different sites, cultivating them on separate plates, and counting and identifying the cultivated microbes.

Interestingly, samples from the drops and, what is more, from the residual interior fluid contained viable bacteria, despite the preservatives present in all of the medications tested in our study. It is true that the antimicrobial efficacy of these substances was not addressed here, but obviously, the concentration of the antiseptics used is too low to inactivate all vegetative forms of pathogens (bacterial spores are naturally resistant) and/or that the inactivation times are long, at least for some strains. For instance, gram-negative bacteria show resistance against quaternary ammonium compounds,^21^ such as benzalkonium chloride, which is generally applied in a range of 0.005% to 0.02%. Actually, preservatives at low concentrations have been reported to be less effective in eyedrops than at higher concentrations.^4,10^ In this regard, it seems logical that antibiotic eyedrops were less frequently contaminated than glaucoma ones in our study despite a similar content of benzalkonium chloride around 0.01%, but this was also the case for anesthetics (Table 1). Notably, all 14 samples of dexamethasone-gentamicin medication were sterile, but also all 29 samples of oxybuprocaine. The latter ones (Novain 0.4%; Agepha Pharma, Vienna, Austria) contain 0.09% chlorhexidine diacetate as a preservative, which is rapidly effective at this concentration.^22^ As preservatives at a too high concentration are toxic for the eye, a balance between toxicity and efficacy has to be found. There is already a trend to single-dose eyedrops especially in glaucoma medication, which is meaningful not only for protection of the ocular surface but also for the reduction of eyedrops contamination.

This study once more indicates the importance of the education of patients and personnel in correct and hygienic application of this kind of medication. The scheme of 1-week use in the operating room seems to be of advantage, though not compared with a longer use at this site. Multiuse eyedrops bottles used daily in the hospital should be capped between applications. The frequent contamination of multidose eyedrops raises the question of whether single-dose eyedrops might be preferred. For estimating a risk of infection, the kind of cultivated pathogen, the number of CFU, and contamination of different sites of the eyedrops should be taken into account.
